# Serum Antioxidative Enzymes Levels and Oxidative Stress Products in Age-Related Cataract Patients

**DOI:** 10.1155/2013/587826

**Published:** 2013-05-28

**Authors:** Dong Chang, Xuefei Zhang, Shengzhong Rong, Qian Sha, Peipei Liu, Tao Han, Hongzhi Pan

**Affiliations:** ^1^Clinical Labororatory, The First Affiliated Hospital of Harbin Medical University, Harbin 150081, China; ^2^Department of Ophtalmology, The Second Affiliated Hospital of Harbin Medical University, Harbin 150081, China; ^3^Public Health School, Mudanjiang Medical College, Mudanjiang 157011, China; ^4^Department of Ophtalmology, Eye Hospital of Heilongjiang Province and the First Affiliated Hospital of Harbin Medical University, Harbin 150081, China; ^5^Department of Nutrition and Food Hygiene, Public Health School of Harbin Medical University, Harbin 150081, China

## Abstract

*Purpose*. To investigate the activity of antioxidative enzymes and the products of oxidative stress in patients with age-related cataracts and compare the findings with those in healthy control subjects. *Method*. Sixty patients with age-related cataract and sixty healthy controls of matched age and gender were included in this study. Serum samples were obtained to detect the antioxidative enzymes of superoxide dismutase (SOD), catalase (CAT), and glutathione peroxidase (GSH-Px), and oxidation degradation products of malondialdehyde (MDA), 4-hydroxynonenal (4-HNE), conjugated diene (CD), advanced oxidation protein products (AOPP), protein carbonyl (PC), and 8-hydroxydeoxyguanosine (8-OHdG). *Results*. Serum SOD, GSH-Px, and CAT activities in cataract group were significantly decreased as compared to the control subjects (*P* < 0.05). The levels of MDA, 4-HNE, and CD in cataract patients were significantly higher than those in the control subjects (*P* < 0.05, *P* < 0.01). Cataract patients had higher levels of 8-OHdG, AOPP, and PC with respect to the comparative group of normal subjects (*P* < 0.01). And there was no statistical significance in concentration of antioxidative enzymes and oxidative stress products in patients with different subtype cataract. *Conclusions*. Oxidative stress is an important risk factor in the development of age-related cataract, and augmentation of the antioxidant defence systems may be of benefit to prevent or delay cataractogenesis.

## 1. Introduction

The age-related cataract is an ever-increasing visual problem that accounts for approximately 50% of blindness worldwide [1]. Epidemiologic studies have indicated that half of the general population older than 65 has cataract [2]. In developing countries, 50–90% of all blindness is caused by cataracts [3]. In the United States more than two million lens extractions are performed annually with the attendant significant health care costs, consuming 12% of the Medicare budget, and a steady increase is projected [4, 5]. Over 50 million people worldwide suffer from cataracts and the number will increase as individuals in the current generation grow older [6, 7]. Currently, there is no effective medical treatment for cataract except surgery. For these reasons, there is much interest in the prevention of cataract as an alternative to surgery. The development of age-related cataract is a slow process; the exact mechanism of cataract formation has not been clearly defined [3]. Multiple mechanisms have been implicated in the development of cataract formation such as excessive tissue sorbitol concentrations, abnormal glycosylation of lens proteins, and increased free-radical production in the intraocular region. They may result in an increasing clouding of the lens until the whole lens loses its normal transparency and becomes white and opaque [8]. There is increasing evidence that oxidative stress has been implicated in the development of age-related cataract. Biochemical evidence demonstrates that the oxidative damage of the lens proteins is involved in the genesis of age-related cataract. In particular, the lens proteins are subjected to extensive oxidative modifications [9–14].

The pathogenesis of cataract is known to be influenced by a number of factors including oxidative stress. Oxidative stress is essentially an imbalance between the production of various reactive species and the ability of the organism's natural protective mechanisms to cope with these reactive compounds and prevent adverse effects. The reactive oxygen species (ROS), which consist principally of molecules like the superoxide anion (O_2_^−^), hydrogen peroxide (H_2_O_2_), and hydroxyl radicals, are detoxified by enzymes such as superoxide dismutase (SOD), catalase (CAT), and glutathione peroxidase (GSH-Px). The antioxidant system and the amount of ROS are kept in a certain state of homeostasis. When exogenous or endogenous factors increase oxidative stress, homeostasis is disturbed and the ROS denature many basic intracellular molecules like nucleic acids, proteins, and lipids. Oxidative stress is widely acknowledged to be a major initiating factor in the development of age-related cataracts [9–14]. 

Taking these studies into account, this study is to evaluate the levels of antioxidative enzymes and oxidative stress products in age-related cataract patients and investigate the relationship between oxidative stress and age-related cataract.

## 2. Materials and Methods

### 2.1. Subjects

Sixty patients with newly diagnosed senile nonpathologic cataract are recruited for the experiment group, among whom 25 patients have cortical cataract, 21 patients have nuclear cataract, and 14 patients have posterior subcapsular cataract. All patients with cataract had severe visual disturbances, and their corrected visual acuities were under 0.3 and had an age-related cataract in at least one eye; they also had no other eye abnormalities that could explain the vision loss. These patients were recruited from the First Affiliated Hospital of the Harbin Medical University and the Eye Hospital of Heilongjiang Province. We excluded patients with secondary cataract due to diabetes, trauma, steroid administration, and other causes. Both groups belonged to the same ethnic group. Sixty healthy, age- and sex-matched subjects were also included for the control. The control subjects were recruited from subjects who came to the same hospital for an annual refractive checkup. The control subjects were in good health, as determined by a medical history questionnaire, physical examination, and normal results of clinical laboratory tests, such as glucose, cholesterol, triglycerides, and blood pressure. None of the study subjects had a history of cardiovascular, hepatic, gastrointestinal, or renal dysfunction; none were alcoholic, smokers; and none used exogenous hormones. Subjects were not permitted to take any supplemental vitamin or carotenoid for more than 6-week before the start of the study and were limited to drinking less than two cups of tea per day during this 6-week period. 

All the subjects underwent a complete ophthalmologic evaluation that included medical history, slit lamp biomicroscopy, Goldmann applanation tonometry, and funduscopy. Ethics Committee of Harbin Medical University has already approved the study protocol. Written informed consent was obtained from each study subject, and all subjects consented to giving blood samples. 

### 2.2. Blood Sampling

Venous peripheral blood samples (10 mL) were collected after 12 h overnight fasting from each subject. The samples were placed on ice and centrifuged for an hour at 3500 rpm, 4°C for 15 min, and the supernatants were stored at −80°C, and determination of the samples occurred within 3 months. All samples from each patient were run in the same assay.

## 3. Laboratory Analysis

### 3.1. Measurement of Activity of Antioxidative Enzymes

SOD activity was determined using the method of Sun et al. [15]. Glutathione peroxidase (GSH-Px) activity was measured by the method of Paglia and Valentine [16]. Catalase (CAT) activity was assayed based on the procedure of Aebi [17]. 

### 3.2. Measurement of Lipid Peroxidation Products

Quantitative estimation of products of lipid peroxidation included assays for conjugated dienes (CD) and malondialdehyde (MDA). CD were extracted from plasma using a 2 : 1 (vol/vol) mixture of chloroform and methanol. Four mL of the chloroform-methanol mixture, preheated to 45°C, was added to 0.1 mL of serum. The mixture was then vigorously mixed (with a vortex machine) for 2 min, then mixed with 2.0 mL of distilled water acidified with 0.1 M HCl to a pH of 2.5. After agitation that used a vortex instrument, the material was subjected to centrifugation (2,000 g for 5 min), and 1.5 mL of the lower layer was aspirated, transferred to a test tube, and dried under a flow of nitrogen gas. The residue was reconstituted with 1.0 mL of heptane and measured spectrophotometrically at 233 nm. 

MDA was measured as thiobarbituric-acid-reacting substance (TBARS) production in the following manner. 0.1 mL of sample was added to a 1 : 1 : 1 (vol/vol/vol) solution of trichloroacetic acid (15%, wt/vol), thiobarbituric acid (0.375%, wt/vol), and hydrochloric acid (0.25 M). The mixture was heated at 100°C for 30 min. The mixture was immediately cooled and then centrifuged (3,500 g for 5 min) to remove undissolved materials. Then the absorbance at 532 nm was determined. The amount of TBARS was calculated from comparison with authentic malondialdehyde.

### 3.3. Measurement of Protein Damage Products

Products of protein damage included advanced oxidation protein products (AOPP) and protein carbonyl content. AOPP were quantified as described by Witko-Sarsat et al. [18]. We placed 200 *μ*L of serum diluted 1 : 5 in phosphate-buffered saline into each well of a 96-well microtitre plate and added 20 *μ*L of acetic acid to each well. For the standards, we added 10 *μ*L of 1.16 M potassium iodide (Sigma, St Louis, MO, USA) to 200 *μ*L of chloramine-T solution (0 to 100 *μ*mol/L) (Sigma, St Louis, MO, USA) in a well and then added 20 *μ*L of acetic acid. The absorbance of the reaction mixture was immediately read at 340 nm against a blank consisting of 200 *μ*L of phosphate-buffered saline, 10 *μ*L of 1.16 M potassium iodide, and 20 *μ*L of acetic acid. AOPP concentrations are expressed as *μ*mol/L of chloramine-T equivalents.

Protein carbonyl (PC) concentrations in plasma were measured by the spectrophotometric assay described by Reznick and Packer [19]. Briefly, to 200 *μ*L plasma, 4 mL of 10 mmol 2,4-dinitrophenylhydrazine (DNPH)/L in 2 mol HCl/L was added. In another tube, 4 mL of 2 mol HCl/L was added to 200 *μ*L plasma (of the same patient). The tubes were left in the dark for 1 h at room temperature and were mixed by vortex every 15 min. Five milliliters of 20% trichloroacetic acid solution was then added to both tubes for a 10 min incubation on ice, after which the tubes were centrifuged (3000 ×g, 5 min, 4°C). The supernatant fluid was discarded and another wash was performed by using 4 mL 10% trichloroacetic acid. The protein pellets were broken mechanically and washed 3 times with ethanol-ethyl acetate to remove free DNPH and lipid contaminants. The final precipitates were dissolved in 2 mL of 6 mol guanidine hydrochloride/L, and the UV absorbance at *λ* = 370 nm was measured spectrophotometrically. The carbonyl content (nmol/mL) was calculated using *ε*_*M*_ = 22,000. 

### 3.4. Measurement of Serum 8-OHdG and 4-Hydroxynonenal

Serum 8-OHdG and 4-HNE were measured with enzyme-linked immunosorbent assay (ELISA) method following the maker's instructions. Quantification of the 8-OHdG and 4-HNE was achieved by comparing the optical densities of each sample to that of an internal standard of known 8-OHdG and 4-HNE at various concentrations.

The assay variances of all methods described above were <10%.

## 4. Other Biochemical Parameters

Blood glucose, triglycerides, and cholesterol were determined using routine clinical chemical assays.

*Statistical Analysis.* Data are presented as mean ± SD. All experimental data in this study were statistically analyzed with SAS 9.13. The statistical significance was evaluated using unpaired Student's *t*-test. Results were considered significant at *P* < 0.05. 

## 5. Results

### 5.1. Baseline Characteristics

The clinical characteristics of the age-related cataract patients and control subjects are shown in Table 1. Age and gender of the patients were not significantly different from those of the controls. Initial clinical laboratory test values, such as glucose, cholesterol, triglycerides, and blood pressure, were also not significantly different between the two groups. Furthermore, the test values were all in normal range.

### 5.2. Results of Activity of Antioxidative Enzymes in Serum

As shown in Table 2, we found the depression of antioxidative enzymes in serum of patients. Compared with the control group, the activities of SOD, GSH-Px, and CAT in cataract group were lower than those in the control group (*P* < 0.05, *P* < 0.01).

### 5.3. Results of Products of Lipid Peroxidation in Serum

As shown in Table 3, there was a significant increase in serum MDA in cataract patients (3.75 ± 1.11 nmol/mL) compared with normal subjects (3.28 ± 1.03 nmol/mL, *P* < 0.05). Serum conjugated dienes in cataract patients have also significantly increased than those in control (0.488 ± 0.125 versus 0.418 ± 0.122, *P* < 0.01). And the concentration of 4-HNE significantly increased in patients' serum compared with normal subjects (15.33 ± 4.63 versus 13.41 ± 3.48, *P* < 0.05).

### 5.4. Results of Protein Oxidative Damage Products in Serum

As shown in Table 4, the increased levels of AOPP as well as protein carbonyl in cataract patients were also observed. The concentration of AOPP in cataract patients was increased significantly than that in control subjects (20.83 ± 5.38 versus 17.81 ± 6.07; *P* < 0.01). Cataract patients had significantly higher protein carbonyl compared with healthy subjects (3.37 ± 1.04 versus 2.90 ± 0.68; *P* < 0.01). 

### 5.5. Results of DNA Damage Products in Serum

There was a significant increase in serum 8-OHdG in cataract patients (22.13 ± 4.80 ng/mL) compared with age-matched normal subjects (6.33 ± 1.73 ng/mL, *P* < 0.01). 

### 5.6. Results of Antioxidative Enzymes in Subtype Cataract Patients

We divided 60 patients with age-related cataract into 3 groups, depending on a type of cataract. The results of antioxidative enzymes in subtype cataract patients were shown in Table 5. There were no statistically significant differences among patients with different subtype of age-related cataract (*P* > 0.05). 

### 5.7. Results of Oxidative Stress Products in Subtype Cataract Patients

We divided 60 patients with age-related cataract into 3 groups, depending on a type of cataract. As shown in Table 6, the results of oxidative stress products in subtype cataract patients showed no statistically significant differences between the three groups (*P* > 0.05). 

## 6. Discussion

Cataract is the major cause of blindness and visual impairment worldwide. According to the anatomical location within the lens, cataract can be subdivided into three subtypes: cortical cataract, nuclear cataract, and posterior subcapsular cataract. The prevalence of the 3 main types of age-related cataracts differs in different regions of the world and in different racial groups. In most tropical areas, nuclear cataracts are the most common. In northern China, cortical cataracts are more prevalent. The incidence of posterior subcapsular cataract is lower than that of nuclear and cortical cataract [20, 21]. Whether these differences are related to differences in genetics, environment, diet, or other factors is difficult to discern; oxidative damage has been implicated as a major contributor to the pathogenesis of age-related cataracts.

Numerous scientific investigations have confirmed the presence of oxidative stress in ocular diseases, and ROS may play a significant role for the pathophysiology in cataracts. ROS have physiological functions at low levels but are toxic to the cell at high levels. To protect against toxic effects of ROS and to modulate physiological effects of ROS, the cell has developed antioxidant defence systems. The systems are very complex, being composed of antioxidative enzymes (such as SOD, GSH-Px, and CAT) and antioxidant compounds (vitamins A, C, E, and so on). SOD decomposes superoxide into hydrogen peroxide. CAT reduces H_2_O_2_ to water. GSH-Px reduces all organic lipid peroxides. They protect cells against ROS produced during normal metabolism and after an oxidative insult. Oxidative stress is defined as a disturbance in the balance between the production of ROS and antioxidant defence systems [22]. ROS is mostly generated within the mitochondria in lens epithelium cells and the superficial fiber cells, which are highly reactive and can damage macromolecules in living cells, such as lipids, proteins, and nucleic acids, causing mutagenesis and cell death [23–25]. 

The lens epithelium cell (LEC) is the center of metabolic activities in lenses, and oxidative damage to LECs plays a significant role in the pathogenesis of many forms of cataracts [14, 25]. At normal conditions, LECs use several strategies to maintain ROS at low levels to protect lipids, proteins, and nucleic acids. These strategies include activation of the ROS scavenger enzymes such as SOD, CAT, GSH-Px, and DNA repair enzymes. These antioxidative enzymes are present in all parts of the lens, which protect the lens from oxidative stress and maintain lens clarity [26, 27]. However, there is a diminution of these ROS scavenger enzymes and decreased DNA repair capability, placing the lens at risk for oxidative damage and cataract [28]. It has been demonstrated that antioxidant enzymes levels are altered in cataracts. Some reports showed that the activity of SOD, GSH-Px, and CAT decreased in cataract [29, 30], but there were other reports with conflicting results [31]. In the present study, we found that there was a significant decrease in the activity of SOD, GSH-Px, and CAT in serum of cataract patients, compared with normal control. But there is no significant difference in subtype cataracts. This result leads us to think that oxidative stress is an important mechanism in the development of cataracts. 

Oxidants are highly reactive compounds with a half-life of only seconds. Therefore, their in vivo determination is generally not feasible. In contrast, lipids, proteins, carbohydrates, and DNA, after being modified by oxyradicals, having lifetimes ranging from hours to weeks, can be measured with biochemical assays, which makes them ideal markers of oxidative stress. Many biomarkers have been developed to evaluate oxidative stress. These markers include lipid peroxidation products (such as acrolein, MDA, CD, and 4-hydroxynonenal), protein oxidation products (such as AOPP, PC), and DNA oxidation products 8-hydroxy-2-deoxyguanosine (8-OHdG). Oxidation of proteins, lipids, and DNA has been observed in cataractous lens [32–34]. 

Oxidation of lipids has been widely studied, and there are more biomarkers available for assessing oxidation of this substrate than for protein and DNA combined. Lipid peroxidation is initiated by free-radical attack of membrane lipids, generating large amounts of reactive products, which have been strongly implicated in the mechanisms of cataractogenesis [35–37]. MDA, 4-HNE, and CD are three markers of lipid peroxidation, widely used in large studies. MDA is a product of the breakdown of mainly unsaturated fatty acids into their essential chains through the oxidation mechanism. 4-HNE derives from *ω*-6 polyunsaturated fatty acids like linoleic and arachidonic acids whose conjugated double bonds are an easy target for species that can extract a hydrogen atom or add to a double bond. CD is the initial formation of a lipid peroxide. Some studies have revealed the increased lipid peroxidation products in human cataractous lenses. Micelli-Ferrari et al. [36] reported that increased levels of MDA were observed in cataractous lenses of cataract patients compared with the control. Babizhayev [37] reported that CD was distinctly accumulated in cataractous lenses of cataract patients compared with the control. In this study, we have demonstrated that the products of lipid peroxidation, MDA, 4-HNE, and CD, increased significantly in serum of cataract patients compare with that of control. Even though the certain mechanisms responsible for lipid peroxidation-mediated cataractogenesis are not clear, the mechanisms for defence against lipid peroxidation can be perceived as one of the major deterrents of cataract caused by the oxidative stress. 

Biomarkers of protein oxidation are often applied when a battery of markers of oxidative stress status is being studied. Several protein modifications may result from oxidative stress and lead to formation of the high-molecular-weight insoluble aggregates that are common in cataractous lenses [38]. The biomarker that is generally used to estimate protein oxidation is protein carbonyl (PC), which is derived from amino acids during metal-catalyzed oxidation of proteins in vitro and in vivo, representing a direct measure of the oxidative injury to these molecules [39]. The concentration of PC was stable, yielded quantitative results, and appeared to reflect disease endpoints in a biologically significant way. Advanced oxidation protein products (AOPP), a new marker of protein oxidation, have begun to attract the attention of various investigators [18, 40, 41]. They are formed during oxidative stress by the action of chlorinated oxidants, mainly hypochlorous acid and chloramines (produced by myeloperoxidase in activated neutrophils). They are elevated in patients with renal insufficiency and diabetes mellitus [42, 43]. In our previous study, we found elevated AOPP in diabetic retinopathy [44]. Elevated markers of protein oxidation have also been associated with cataractogenesis, [45]. In the present study, we have also determined the level of AOPP and protein carbonyl, two products of protein oxidation, which were increased, and demonstrated that there was protein oxidative damage in cataracts. To the best of our knowledge, no studies have identified the relationship between AOPP and cataracts. Our results show that AOPP is a biomarker available for assessing oxidative stress in age-related cataracts. 

DNA bases are very susceptible to ROS oxidation; the most commonly used biomarker for assessing oxidative DNA damage is 8-hydroxy-2-deoxyguanosine (8-OHdG). DNA can be oxidized to produce many oxidative products; however, oxidation of the C-8 of guanine is one of the most common oxidative events and results in a mutagenic lesion that produces predominantly G-to-T transversion mutations. 8-OHdG was found to be increased in a normal human LEC culture after induced oxidative stress [46]. Ates et al. [47] confirmed a significantly higher level of leukocyte 8-OHdG in the patients with cataract than in the control patients. Some studies have showed association of DNA damage with human cataracts [25, 48]. One important study showed association of DNA damage with human cataracts [33]. In another study, Sorte et al. [49] found that there was significant DNA damage in LECs of senile cataract patients. Moreover, DNA damage in cortical cataracts was significant when compared to that of nuclear or posterior subcapsular cataracts, but the DNA damage between nuclear and posterior subcapsular cataracts was not significant. In our study, we found an overall elevation of serum 8-OHdG in patients with cataract, but there were no statistically significant differences among patients with different type of age-related cataract. These data suggest that 8-OHdG levels are a potentially useful marker of oxidative DNA damage in cataract patients. 

## 7. Conclusions

In the present study, we found that there is a significant disequilibrium status of antioxidative systems in serum in the age-related cataract patients. Compared with the control group, the activities of SOD, GSH-Px, and CAT in cataract group were lower than those in the control group and the oxidative stress products MDA, 4-HNE, CD, AOPP, PC, and 8-OHdG were significantly increased in serum in cataract patients. But there was no statistically significant difference among patients with different subtype of age-related cataract. Our results obtained here confirm that oxidative stress, present or initiating factor in all three types of cataract, is involved in the development of cataract, and augmentation of the antioxidant defences may be helpful to prevent or delay cataractogenesis.

## Figures and Tables

**Table 1:** Clinical characteristics of age-related cataract patients and control.

Demographics	Health group	Cataract patients
*N*	60	60
Age (years)	58.9 ± 8.4	61.0 ± 10.3
Sex (F/M)	30/30	32/28
Cholesterol (mmol/L)	5.01 ± 0.69	4.97 ± 0.63
Triglycerides (mmol/L)	1.16 ± 0.38	1.14 ± 0.32
Glucose (mmol/L)	4.65 ± 0.53	4.68 ± 0.70
Systolic blood pressure (mmHg)	126.4 ± 6.6	125.5 ± 6.7
Diastolic blood pressure (mmHg)	78.9 ± 5.1	78.2 ± 6.4

Data are means ± SD.

**Table 2:** Serum activity of SOD, GSH-Px, and CAT.

Group	SOD (U/mL)	GSH-Px (*μ*mol/L)	CAT (U/mL)
Control	103.47 ± 18.97	147.90 ± 20.21	5.75 ± 1.30
Cataract	97.26 ± 13.56*	135.75 ± 32.45*	5.05 ± 1.46**

Data are means ± SD. *P < 0.05 versus healthy subject, **P < 0.01 versus healthy subject.

**Table 3:** Serum levels of MDA and conjugated dienes.

Group	MDA (nmol/mL)	4-HNE (nmol/mL)	Conjugated dienes (OD, 233 nm)
Control	3.28 ± 1.03	13.41 ± 3.48	0.418 ± 0.122
Cataract	3.75 ± 1.11*	15.33 ± 4.63*	0.488 ± 0.125**

Data are means ± SD. *P < 0.05 versus healthy subject, **P < 0.01 versus healthy subject.

**Table 4:** Serum levels of AOPP and protein carbonyl.

Group	AOPP (*μ*mol/mL)	Protein carbonyl (nmol/mL)
Control	17.81 ± 6.07	2.90 ± 0.68
Cataract	20.83 ± 5.38**	3.37 ± 1.04**

Data are means ± SD. **P < 0.01 versus healthy subject.

**Table 5:** The results of antioxidative enzymes in subtype cataract patients.

	Cortical cataract	Nuclear cataract	Posterior subcapsular cataract
SOD (U/mL)	98.05 ± 12.35	96.31 ± 15.65	97.26 ± 13.17
GSH-Px (U/mL)	130.52 ± 33.60	140.57 ± 30.97	137.88 ± 33.54
CAT (U/mL)	4.93 ± 1.43	5.15 ± 1.50	5.09 ± 1.56

Data are means ± SD.

**Table 6:** The results of oxidative stress products in subtype cataract patients.

	Cortical cataract	Nuclear cataract	Posterior subcapsular cataract
MDA (U/mL)	3.57 ± 1.22	3.75 ± 1.12	4.06 ± 0.89
CD (OD, 233 nm)	0.496 ± 0.127	0.511 ± 0.119	0.440 ± 0.129
4-HNE (nmol/mL)	15.20 ± 5.00	15.18 ± 3.47	15.78 ± 5.68
AOPP (U/mL)	20.66 ± 6.51	21.27 ± 4.69	20.50 ± 4.33
PC (mg/L)	3.40 ± 1.11	3.14 ± 0.93	3.63 ± 1.06
8-OHdG (ng/mL)	21.88 ± 5.09	23.04 ± 4.17	21.22 ± 5.24

Data are means ± SD.
